# VENTILA2: A Fuzzy Logic-Based Simulation and Decision-Support Framework for Pressure-Controlled Ventilation—A Proof of Concept

**DOI:** 10.3390/healthcare14182925

**Published:** 2026-09-09

**Authors:** Lucas Carrera-Villar, Julia López-Canay, Jaime Álvarez-Vázquez, Manuel Casal-Guisande, María Torres-Durán, Alberto Fernández-Villar

**Affiliations:** 1 NeumoVigo I+i Research Group, Galicia Sur Health Research Institute (IIS Galicia Sur), SERGAS-UVIGO, 36312 Vigo, Spain; lucas.carrera@iisgaliciasur.es (L.C.-V.); julia.lopez@iisgaliciasur.es (J.L.-C.); maria.luisa.torres.duran@sergas.es (M.T.-D.); alberto.fernandez.villar@sergas.es (A.F.-V.); 2 Fundación Pública Galega de Investigación Biomédica Galicia Sur, Hospital Álvaro Cunqueiro, 36312 Vigo, Spain; 3 School of Industrial Engineering, University of Vigo, 36310 Vigo, Spain; 4 Department of Design in Engineering, University of Vigo, 36208 Vigo, Spain; 5 Centro de Investigación Biomédica en Red, CIBERES ISCIII, 28029 Madrid, Spain; 6 Pulmonary Department, Hospital Álvaro Cunqueiro, 36312 Vigo, Spain; 7 Department of Functional Biology and Health Sciences, University of Vigo, 36310 Vigo, Spain

**Keywords:** non-invasive mechanical ventilation, simulation platform, decision support system, chronic obstructive pulmonary disease, acute respiratory distress syndrome, fuzzy logic

## Abstract

**Background and Objectives**: Non-invasive mechanical ventilation is the first-line treatment for managing acute respiratory failure. However, patient variability and complex pulmonary mechanics complicate therapy adjustments, frequently leading to ventilator-induced lung injuries. This study aims to propose and define a simulation platform and decision support prototype, named VENTILA2, to optimize pressure-controlled ventilation strategies. **Methods**: The system integrates a bicompartmental series model of the respiratory system incorporating severity-stratified physiological profiles of chronic obstructive pulmonary disease and acute respiratory distress syndrome, and it is coupled with a Mamdani fuzzy inference system. This architecture maps inspiratory time adjustments based on pressure errors and their derivatives across predefined clinical profiles within a scenario-based feedforward parameter-mapping framework. **Results**: Evaluated through quantitative operational verification across all profiles and proof-of-concept case studies, the platform successfully recreates complex clinical scenarios, accurately simulating phenomena such as accelerated lung emptying in severe acute respiratory distress syndrome and air trapping in moderate chronic obstructive pulmonary disease. **Conclusions**: VENTILA2 provides a controlled simulation environment for evaluating pathology-specific ventilatory configurations across simulated profiles prior to clinical implementation, though it remains an early-stage prototype whose clinical effectiveness, safety, and robustness remain to be rigorously evaluated.

## 1. Introduction

Non-invasive mechanical ventilation (NIV) is the first-line treatment for acute respiratory failure. However, managing patient–ventilator interactions is complex, and improper parameter selection directly threatens patient safety [1]. Suboptimal configurations frequently cause ventilator-induced lung injuries like barotrauma or volutrauma [1]. Therefore, performing adequate configurations based on dynamic characteristics of a patient’s pulmonary mechanics is key to preventing patient–ventilator asynchronies and effectively reducing the complication rate associated with artificial life support [1].

The need for therapeutic individualization takes on relevance given the high global prevalence of respiratory diseases. It is estimated that chronic obstructive pulmonary disease (COPD) affects approximately 10.3% of the global population and currently ranks as one of the top three causes of death worldwide [2,3]. Meanwhile, in the intensive care setting, acute respiratory distress syndrome (ARDS) represents a major public health challenge, affecting 10% of all patients admitted to intensive care units, within a context where 30% to 50% of critically ill patients require some form of mechanical ventilatory support [1].

In this scenario, the development of in silico models of the human respiratory system allows for the analysis of pulmonary pathophysiology and the predictive evaluation of ventilatory strategies [1]. These models frequently simulate biological behavior based on electrical circuit analogies, where physiological variables such as pressure, volume, and flow are represented by voltage, charge, and current. They use resistances and capacitances to emulate airway flow resistance and pulmonary compliance, respectively [4,5]. Mathematical modeling allows for the design, optimization, and validation of clinical configurations. It enables therapies to be tested safely on virtual cohorts without risking patient safety [1,6].

The scientific literature describes a constant evolution from linear single-compartment models, which are effective for simulating spontaneous resting breathing, to more complex nonlinear models that incorporate variable resistances and static and dynamic compliances to reflect the variable behavior of the respiratory tract [5,7]. For instance, the single-compartment model simplifies global mechanics through a single chamber (resistor and capacitor in series), the bicompartmental model segments the airway into two branches (series or parallel) to capture the heterogeneity of alveolar regions more realistically, and some complex models include inertia as inductances, which emulate transient phenomena in high-frequency ventilation [5].

The MATLAB/Simulink environment [8] and physical simulation tools like Simscape have become the primary computational environments due to their versatility and the availability of specific blocks for the mathematical modeling of the lung and ventilator [9,10]. A notable example is the work by Jaber et al. [11], who developed a computational model on this platform that couples a pressure-controlled ventilation signal to single and bicompartmental lung models. This setup enables the dynamic monitoring of critical variables such as volume, flow, and pressure–volume loops in response to variations in positive end-expiratory pressure (PEEP) [11].

From a control perspective, fuzzy logic has proven to be highly efficient in representing the non-stationary and imprecise nature of biological systems [4,12]. The work of Güler and Ata [4] stands out in this field for designing a fuzzy logic-controlled mechanical ventilator in Simulink capable of independently estimating inspiratory and expiratory times [4,9]. Despite these advancements, a large portion of current models in the literature is limited to studying a healthy lung archetype or analyzing a single pathology [9].

Within this context, integrating computational models with advanced control strategies permits the evolution from simple simulation to the development of clinical decision support systems (CDSSs) [13]. These are frequently defined as technological tools designed to provide healthcare professionals with evidence-based recommendations and patient-specific data [13], although the present prototype generates synthetic data to assess individual ventilation strategies.

The main purpose of this work is to design and develop a simulation platform and decision support prototype focused on pressure-controlled ventilation. To achieve this goal, we adopted an approach based on fuzzy logic that mimics clinical reasoning by adaptively mapping the inspiratory time (*T*_i_) according to expert rules. The platform we created, named VENTILA2, performs this parameter mapping across preconfigured profiles, allowing for optimized strategies before implementing them in clinical practice.

Our article introduces the novelty of incorporating multiple respiratory profiles (healthy and pathological, representing COPD and ARDS across different severity levels) within a patient model coupled with a pressure-regulated fuzzy logic controller. The specific contributions of this research are as follows:

- Development of a simulation platform for pressure-controlled mechanical ventilation strategies optimization: VENTILA2 is presented as an innovative platform that enables the predictive exploration of various ventilatory configurations within a secure virtual environment.
- Fuzzy logic integration: Our system incorporates a Mamdani-type fuzzy inference system (FIS) that maps *T*_i_ adjustments according to the preconfigured mechanical requirements of each simulated respiratory profile within a feedforward parameter-mapping architecture, emulating expert clinical reasoning.
- Proof-of-concept evaluation: Our system has been evaluated by simulating complex clinical scenarios (COPD and ARDS), demonstrating its capability to display the patient’s response to different degrees of pathology. This approach allows for the safe evaluation of pathology-specific ventilatory strategies across simulated clinical profiles in a preclinical setting.

The intended end users of the VENTILA2 platform comprise respiratory therapists and intensivists operating within intensive care units (ICUs) and subintensive care settings such as intermediate respiratory care units (IRCUs).

The end purpose of the system, prior to clinical validation, is to serve as a preclinical proof-of-concept simulation tool for evaluating mechanical ventilation strategies. It assists users by providing interpretable, rule-based computational outputs that demonstrate how the fuzzy inference system maps *T*_i_ adjustments across selected synthetic respiratory profiles.

The article is organized into four sections. Section 1 introduces the problem and defines the framework of the proposed system. Section 2 details the methods used, beginning with the conceptual design of the intelligent system (Section 2.1) and its subsequent implementation in the MATLAB/Simulink (R2023a version, Natick, MA, USA) environment [8] (Section 2.2). Section 3 presents two practical case studies alongside a quantitative operation verification across all simulated profiles to evaluate the application’s performance as a proof of concept. Lastly, Section 4 presents the discussion of the work, analyzing its limitations against the existing literature, and outlines the main conclusions alongside future lines of research.

## 2. Materials and Methods

### 2.1. Conceptual Design

**Figure 1:**
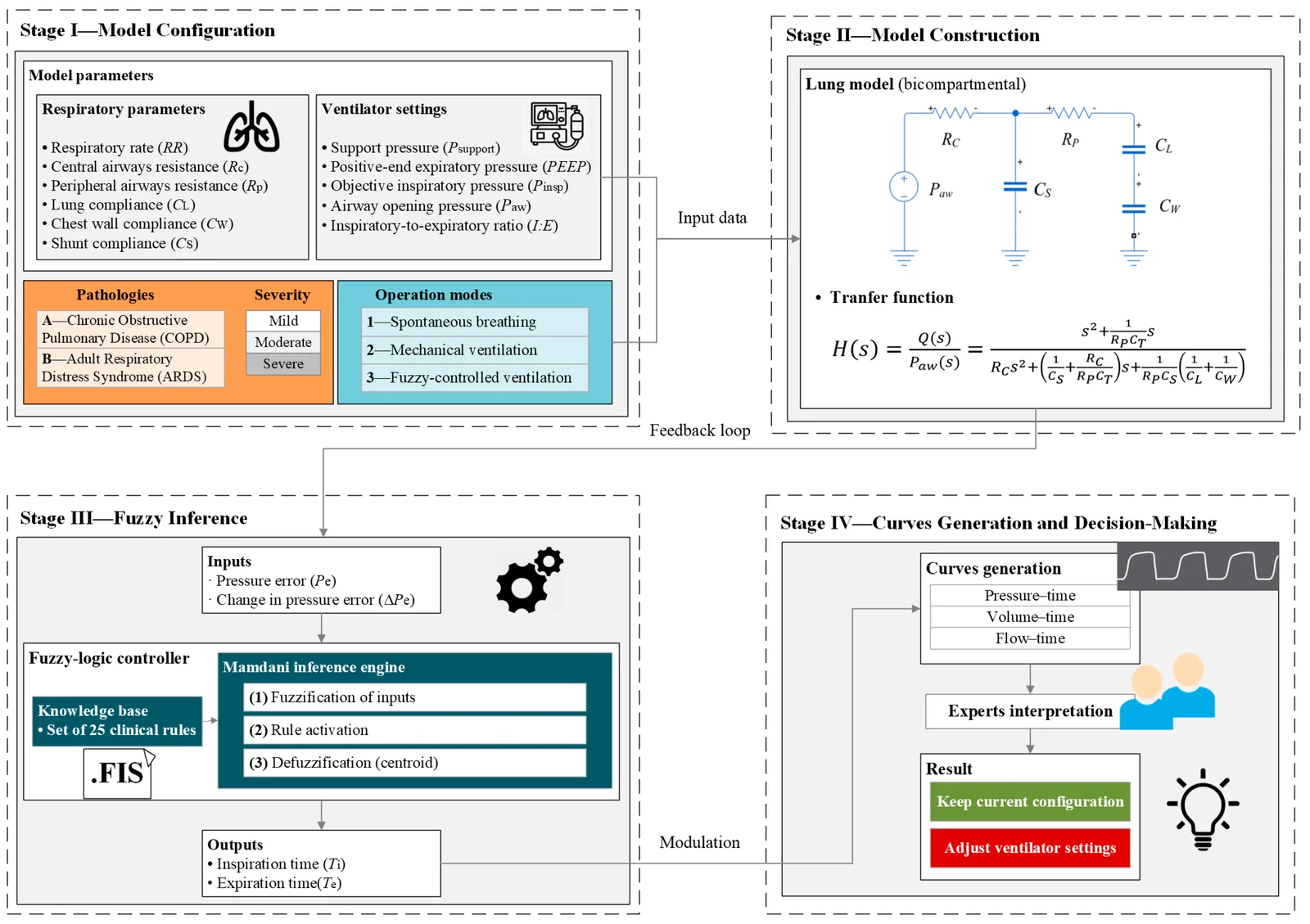
Flow diagram of the proposed system, VENTILA2. Note that the decision-making outputs in Stage IV (“keep current configuration” versus “adjust ventilator settings”) reflect the clinician’s independent evaluation of the visual curves rather than automated system directives.

Figure 1 shows the flow diagram of the VENTILA2 system, which presents a modular architecture structured into four sequential stages listed below.

- Stage I involves the physiological characterization of the synthetic patient through the parametrization of the lung parenchyma mechanical properties and the initial ventilator setup. It includes three operating modes and seven respiratory profiles including healthy, COPD, and ARDS individuals stratified by severity level (mild, moderate and severe).
- Stage II describes the construction of the respiratory mechanics model, defined by its transfer function, *H*(*s*).
- Stage III incorporates a FIS that maps *T*_i_ according to the specific patient requirements.
- Stage IV yields the patient’s response visible in the output respiratory curves to facilitate clinical interpretation and decision making.

Each of these stages is described in further detail in the following subchapters.

#### 2.1.1. Stage I: Model Configuration

The first phase of the system consists of establishing the boundary conditions and variables for both the synthetic patient and the ventilator device. The input module allows for simulation based on preconfigured clinical profiles or manually introduced customized parameter values. This versatility allows for precise adaptation to specific research needs.

- Regarding the operating mode selection, the system integrates three distinct operating modes, which determine the nature of the airway pressure (*P*_aw_) signal applied to the model. Table 1 describes the physiological or mechanical logic underlying each ventilation strategy, from the simulation of basal spontaneous breathing (using a sinusoidal wave) to fuzzy logic-controlled ventilation, employing a pulse-width modulation signal (PWM).

**Table 1:** Operating modes.

Operating Mode	Description
1—Spontaneous breathing	It corresponds to the expiration of an average person at rest without ventilatory support. The pressure signal is approximated by a sinusoidal wave.
2—Mechanical ventilation	It is typical of patients on NIV. The remarkable oscillations of the pressure waveform typical of controlled breathing are approximated by a square wave within a significant margin of error.
3—Fuzzy-controlled ventilation	It enables the FIS, which maps *T*_i_ adjustments according to the measurement of pressure error and its derivative.

The ventilation control variables required for physiological modeling and ventilator regulation are shown in Table 2. Their specification is essential to generate the patient’s pressure waveform in Stage IV.

The respiratory rate (*RR*) establishes the periodicity and baseline of the airway pressure signal. Meanwhile, positive end-expiratory pressure (*PEEP*) ensures proper signal amplitude modeling during non-invasive ventilation to maintain therapeutic levels.

Nevertheless, the pressure-controlled ventilation strategy implemented in Mode 3 requires another variable to regulate the system, which is the target inspiratory pressure (*P*_insp_). This is reference for the FIS’s operation, which permits the inference algorithm to evaluate the pressure error and map *T*_i_ adjustments necessary to reach the desired setpoint.

**Figure 2:**
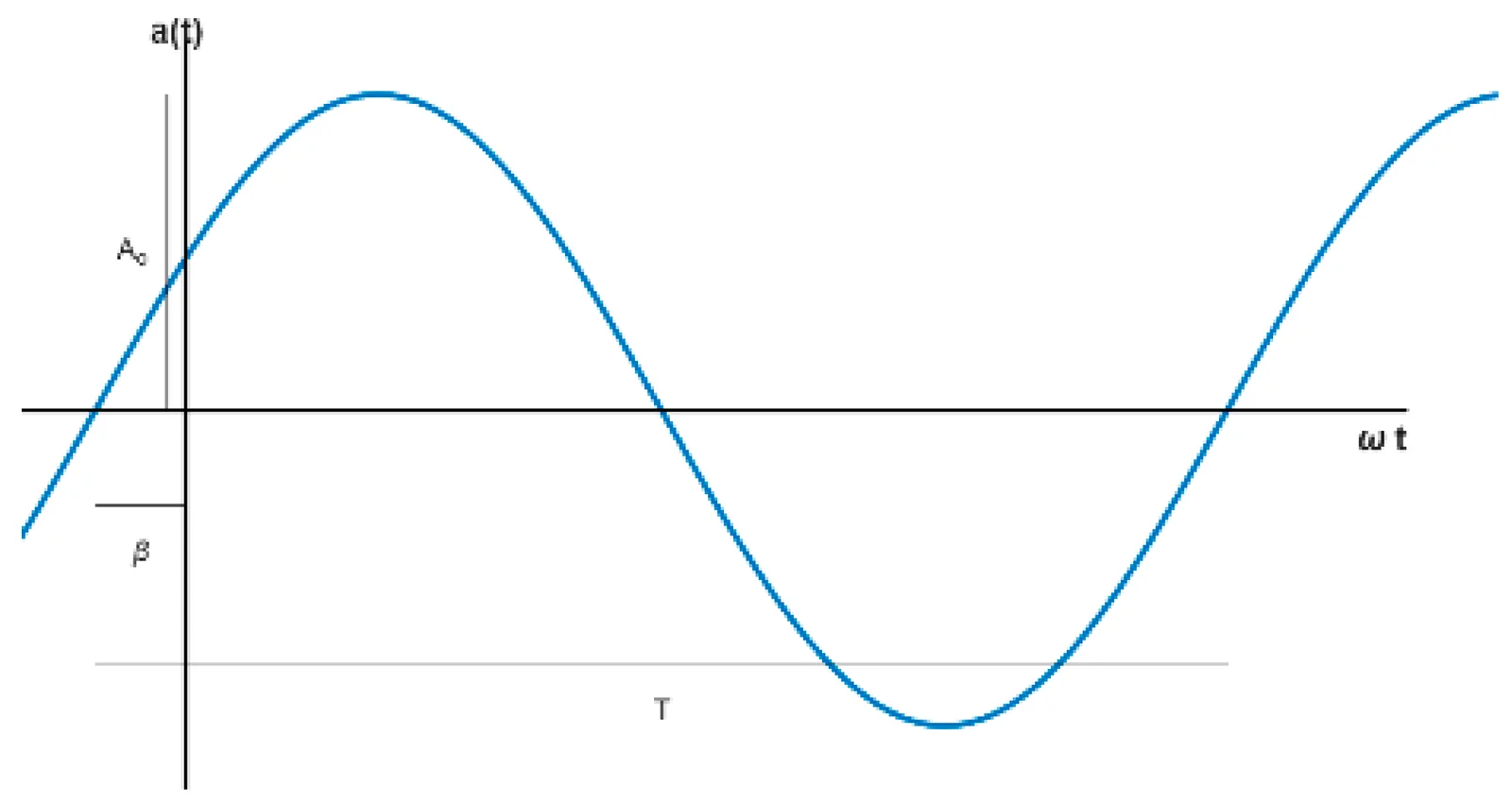
Sinusoidal wave with period parameter *T* and zero offset *β* for the present application due to the simplification of asynchronies. The angular frequency is given by ω and the maximum amplitude by *A*_0_.

The *P*_aw_ waveform is modeled in three ways depending on the operating mode selected from Table 1 and based on the parameters in Table 2. In the case of Mode 1 for spontaneous breathing, a sinusoidal wave with amplitude *A* and period *T* is used, as shown in Figure 2, where the positive half-cycles represent the inspiratory phases and the negative half-cycles represent the expiratory phases. The sinusoidal wave is expressed by (1), where the angular frequency (*ω*) is obtained from the respiratory rate by applying (2). The offset of the wave is determined by *β*, and *A*_0_ dictates its maximum amplitude.

(1) $$P_{aw}\left(t\right)=A_{0}\cdot sin(\omega t+\beta )$$

(2) ω=RR×(2π/60)

**Figure 3:**
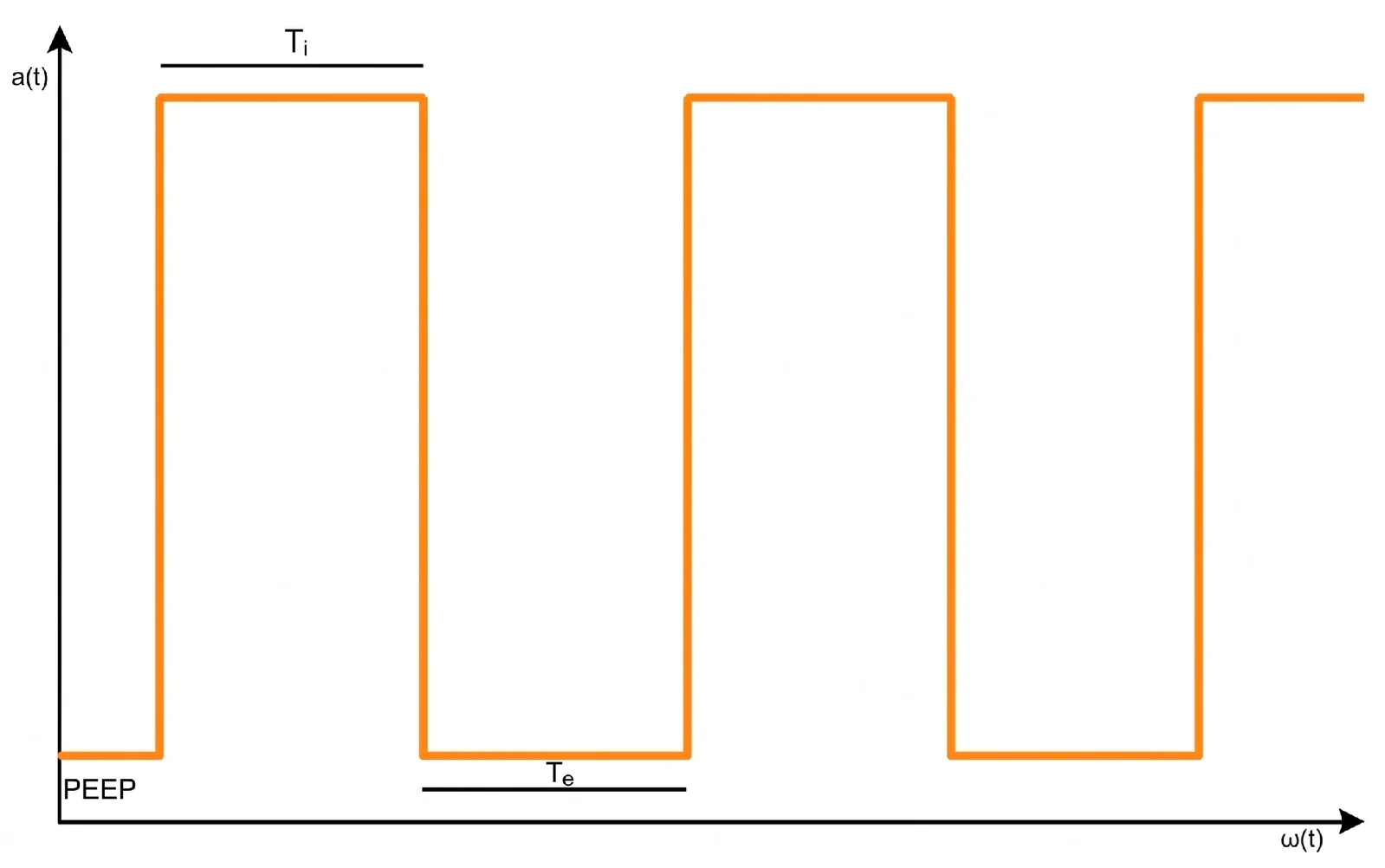
Square wave with parameters for *T*_i_, expiratory time (*T*_e_), and *PEEP*. The signal period is given by the total respiratory cycle time (*TCT*).

For Modes 2 and 3, which involve controlled ventilation simulating interaction with a mechanical ventilator, the waveform is approximated by a square wave, where the high state (“1”) corresponds to the inspiratory phase, while the low state (“0”) represents the expiratory phase. This is a simplification of a real-world pressure waveform, which typically has a trapezoidal shape because the system requires a certain amount of time to reach *P*_insp_ [11]. However, in this present work, as in the article by Jaber et al. [11], an immediate transition between states is considered for the sake of simplification (Figure 3).

For both the square and sinusoidal signals, the signal period or total respiratory cycle time (*TCT*) spans each pair of inspiratory and expiratory phases and can be calculated based on the respiratory rate in breaths/minute, following (3). Note that the baseline temporal formulation in (3) governs the fixed, open-loop timing structures applied in Modes 1 and 2. Furthermore, the inspiration-to-expiration ratio (*I*:*E*) determines the proportion between *T*_i_ and the expiratory time (*T*_e_) according to (4). This is a critical parameter in NIV, as it has a direct impact on the patient’s clinical safety [10].

(3) $$TCT=\frac{60}{RR}$$

(4) $$I:E=\frac{T_{i}}{T_{e}}$$

Equation (4) plays a crucial role in the fuzzy inference of Mode 3, as *T*_e_ is dynamically adapted by the controller based on the fuzzy-inferred *T*_i_ and the prescribed *I*:*E* ratio.

Regarding the amplitude of the square pressure signal, the maximum value corresponds to the sum of two pressures: on one hand, the support pressure (*P*_support_) and, on the other, the *PEEP* introduced by the ventilator, according to (5) [11].

(5) $$P_{aw}\left(t\right)=\left\{\begin{array}{c} PEEP+P_{support},\;\;\;if\;0<t<T_{i}\\ PEEP,\;\;\;\;\;\;\;\;\;\;\;\;\;\;\;\;{if\;\;T}_{i}<t<TCT \end{array}\right.$$

The platform allows for the re-creation of seven predefined physiopathological scenarios, including healthy respiratory profile and ARDS and COPD synthetic patients stratified by levels of severity. The clinical severity categories for both ARDS and COPD across the platform span from mild to severe tiers, aligned with standard clinical classification frameworks [3,14].

#### 2.1.2. Stage II: Model Construction

**Figure 4:**
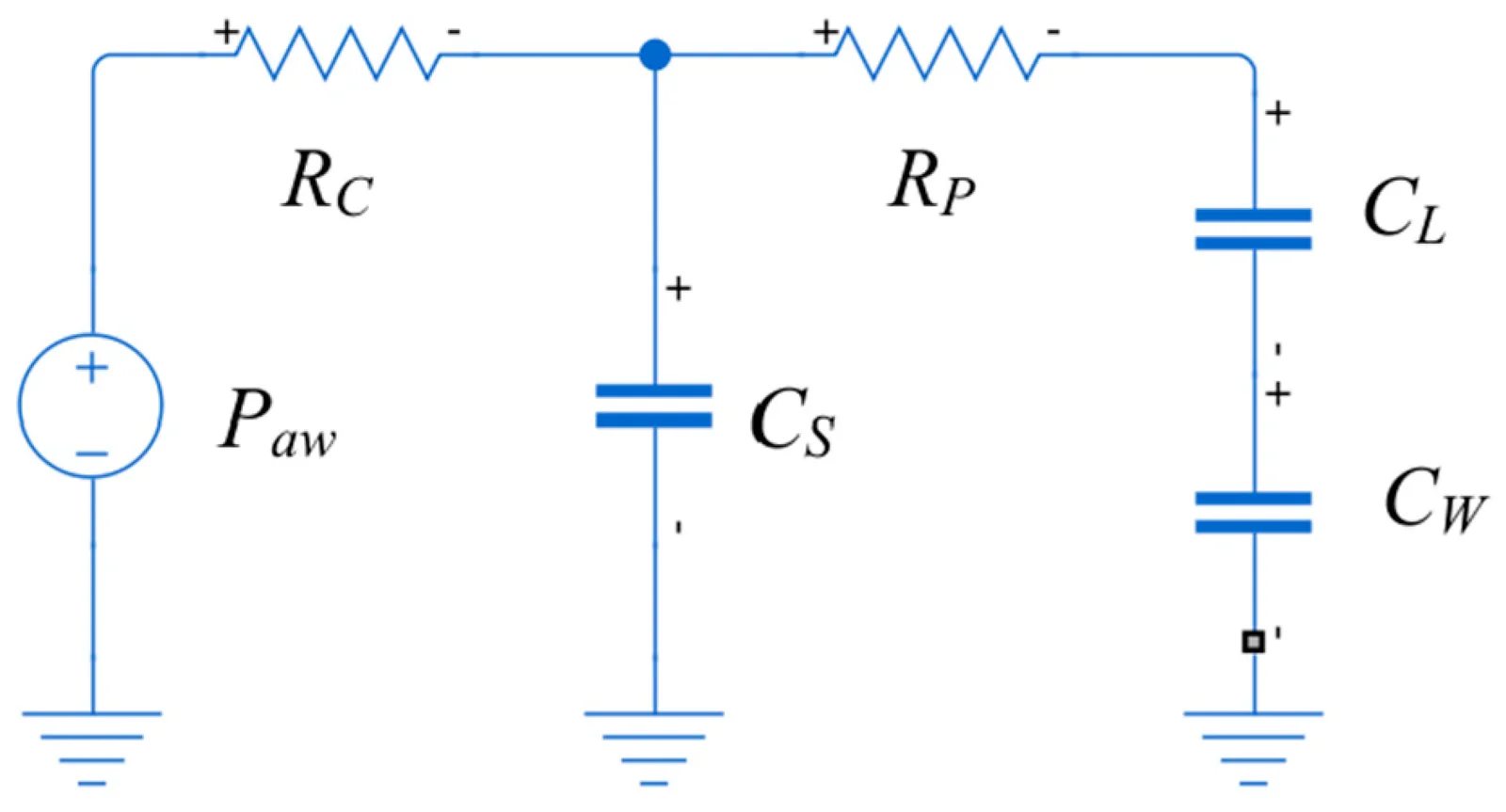
Bicompartmental series model.

Once the input parameters have been defined, it is necessary to establish the mathematical model that represents the global dynamics of the respiratory system, the parameters of which are indicated in Table 3 [4,5]. We implemented a two-compartment series model (Figure 4) that segments the airway into two branches to account for regional heterogeneity without unnecessarily increasing computational cost [5].

The resistive compartments correspond to the central airway (trachea and main bronchi, represented by resistance *R*_C_) and the peripheral airway (bronchioles and alveoli, where gas exchange occurs, with resistance *R*_P_). The volumetric expansion of the system in response to *P*_aw_ is dictated by the patient’s elastic properties, represented by the series interaction of the lung compliance (*C*_L_) and the chest wall compliance (*C*_W_). Based on the series combination of both, the total compliance (*C*_T_) of the system is defined by (6) [15].

(6) $$\frac{1}{C_{T}}=\frac{1}{C_{L}}+\frac{1}{C_{W}}$$

The shunt compliance (*C*_S_) does not correspond to a specific, isolated anatomical zone of gas exchange. Instead, it models the elastic properties of the larger conducting airways (proximal airway compliance) combined with the physical compressibility of the gases within the respiratory system [5,15].

The transfer function (7) models the mechanical properties of the emulated respiratory profile, *Q*(*s*) being the airflow exchange in L/s [4]. This function represents the global dynamics of the patient’s respiratory system and is a function of the properties of the pulmonary parenchyma (resistances and compliances) of the simulated subject, reviewed in Table 3.

(7) $$H\left(s\right)=\frac{Q(s)}{P_{aw}(s)}=\frac{s^{2}+\frac{1}{R_{P}C_{T}}s}{R_{C}s^{2}+\left(\frac{1}{C_{S}}+\frac{R_{C}}{R_{P}C_{T}}\right)s+\frac{1}{R_{P}C_{S}}\left(\frac{1}{C_{L}}+\frac{1}{C_{W}}\right)}\;$$

Equation (7) is obtained from the bicompartmental model’s differential Equation (8) applying the Laplace transform [4].

(8) $$\ddot{P_{aw}}(t)+\frac{1}{R_{P}C_{T}}\dot{P_{aw}}\left(t\right)=R_{C}\cdot \ddot{Q}(t)+\left(\frac{1}{C_{S}}+\frac{R_{C}}{R_{P}C_{T}}\right)\dot{Q}(t)+\frac{1}{R_{P}C_{S}}\left(\frac{1}{C_{L}}+\frac{1}{C_{W}}\right)Q(t)$$

To evaluate the system’s behavior under different clinical scenarios, the model parameter values have been established in Table 4 and were cross-referenced with the specific literature on computational respiratory modeling and pathophysiology [16]. It is important to clarify that these severity strata represent mechanical archetypes defined strictly by specific compliance and resistance alterations rather than complete diagnostic classifications (such as the Berlin definition for ARDS or GOLD grades for COPD [3]).

In this framework, COPD is simulated by an increase in peripheral airway resistance (*R*_P_), which emulates chronic airflow limitation and the intrinsic narrowing of the airways. Conversely, ARDS profiles are constructed through a drastic reduction in *C*_L_, which reflects parenchymal stiffness, edema, and decreased aerated lung volume characteristic of this inflammatory injury, while keeping resistances at more conservative thresholds [17,18].

As a methodological simplification for this initial proof-of-concept framework, *R*_C_, *C*_W_, and *C*_S_ were maintained at fixed nominal values (1.00, 0.20, and 0.005, respectively) to isolate the primary parenchymal mechanics, though we acknowledge that these secondary parameters also exhibit real-world inter-patient variability [16].

These profiles establish the initial conditions that the intelligent system will process to module the therapy.

#### 2.1.3. Stage III: Fuzzy Inference

The model incorporates a FIS that maps *T*_i_ adjustments according to the preconfigured mechanical requirements of each simulated respiratory profile. It operates as a feedforward control system that performs this time modulation as an output variable based on two input variables [4]:

- Pressure error (*P*_e_): Defined as the difference between the target inspiratory pressure (*P*_insp_) and the measured pressure (*P*_meas_) at instant *k*, according to (9).
- Change in pressure error (Δ*P*_e_): This variable represents the rate of change in the error between two consecutive sampling instants obtained by dividing the pressure error difference by the discrete sample time (*T*_s_), as shown in (10).

(9) $$P_{e}(k)=P_{insp}(k)-P_{meas}(k)\;$$

(10) $$\Delta P_{e}(k)=\frac{P_{e}\left(k\right)-P_{e}(k-1)}{T_{s}}\;$$

From a control standpoint, the intelligent controller aims to support ventilation strategies, facilitating the optimization of patient–ventilator interaction.

For the design of the Mamdani-type FIS [19], triangular membership functions (MFs) are employed. The following linguistic labels are used to map both numerical inputs into fuzzy sets [4]:

- NB (Negative Big): Represents a significant negative deviation from the target.
- NS (Negative Small): Represents a minor negative deviation.
- Z (Zero): Indicates the measured value is effectively equal to the target.
- PS (Positive Small): Represents a minor positive deviation.
- PB (Positive Big): Represents a significant positive deviation.

**Figure 5:**
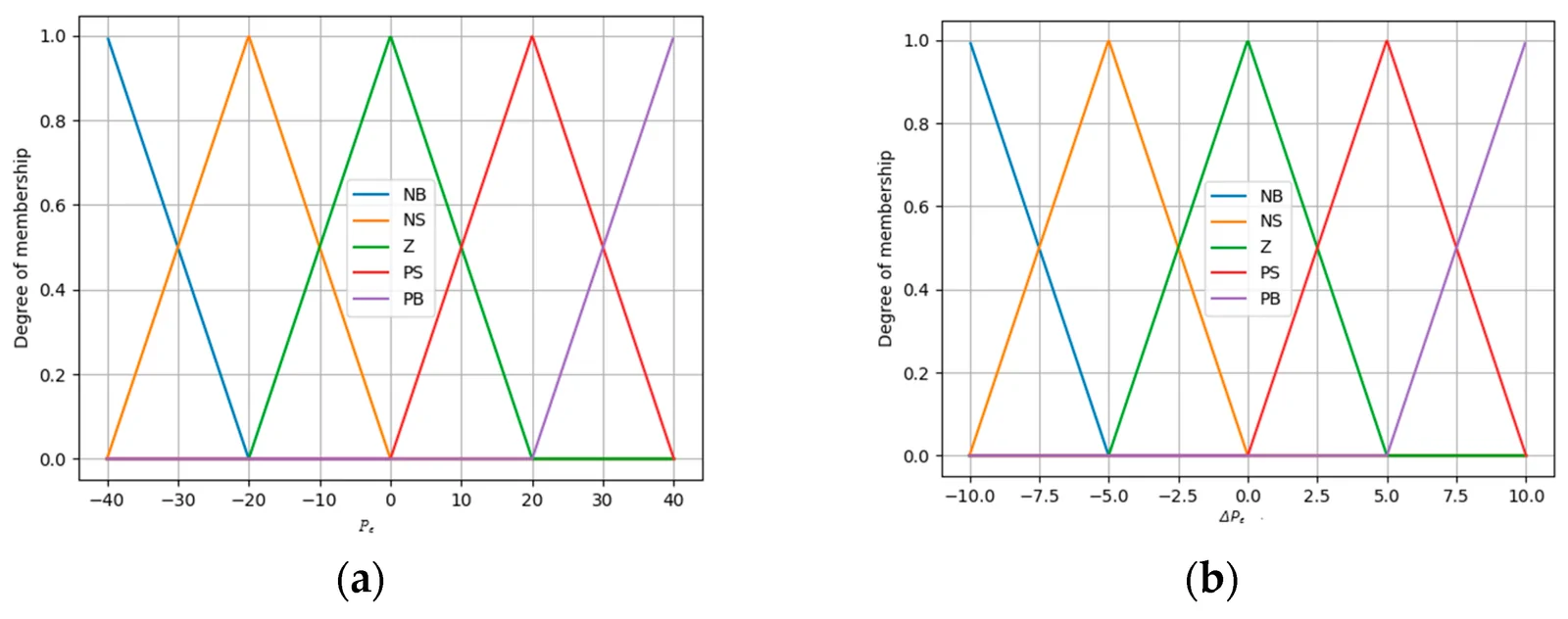
(**a**) Membership functions of the first antecedent (*P*_e_); (**b**) membership functions of the second antecedent (Δ*P*_e_).

**Figure 6:**
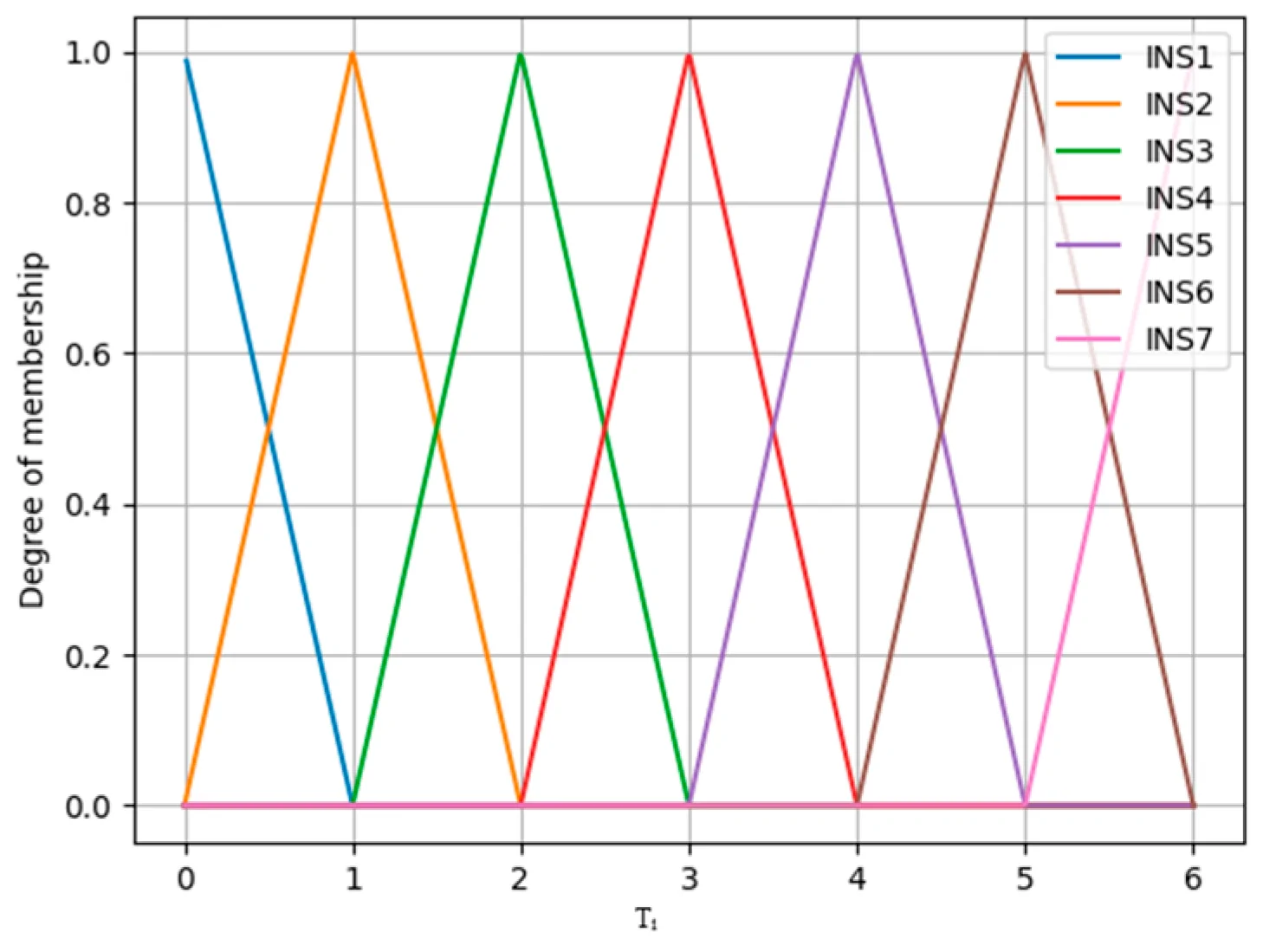
Membership functions of the consequent (*T*_i_).

The choice of triangular functions over other types (such as trapezoidal or Gaussian) is based on their computational efficiency in representing precise transitions and their suitability for real-time clinical applications requiring straightforward interpretability [20,21,22]. Figure 5 shows the triangular activation functions for both the antecedents (*P*_e_ and Δ*P*_e_), while Figure 6 shows those of the consequent (the adjusted *T*_i_) along with their respective value ranges implemented in the MATLAB Fuzzy Logic Toolbox [23].

The 25-rule knowledge base (shown in Table 5) and triangular MF were adopted from the foundational work of Güler et al. [4] as an initial reference for constructing the fuzzy inference component. To enhance reproducibility, the exact breakpoints defining these triangular MFs are explicitly integrated into Table 5, including those of the two antecedents (*P*_e_ and Δ*P*_e_), and Table 6, including the consequent (*T*_i_) MF. The first antecedent (*P*_e_) spans a universe of discourse from −40 to +40 cmH_2_O; the second antecedent (Δ*P*_e_) spans from −10 to +10 cmH_2_O/s, and the consequent (*T*_i_) spans from 0 to 6 s.

Formal quantitative sensitivity analyses evaluating alternative boundary ranges were beyond this prototype’s scope. Furthermore, these rules and boundaries were not optimized for individual COPD or ARDS severity profiles; all rules are conjunctive and share equal weight, ensuring that the influence of each rule on the output is identical.

For instance, if the *P*_e_ is NS and the Δ*P*_e_ is Z, the system evaluates the intersection (using the AND operator) of the corresponding rules in Table 7 to determine the firing strength for the INS3 output MF. This process is repeated for all active rules, and the final crisp value is computed using the centroid of the aggregated membership area.

Although the fuzzy inference system modulates inspiratory time based on pressure errors, the framework operates via scenario-based physiological archetypes rather than instantaneous, real-time dynamic parameter identification of individual patient mechanics.

#### 2.1.4. Stage IV: Curves Generation and Decision Making

The final stage of the system involves the real-time resolution of the mathematical model and the visual representation of pulmonary responses. In the FIS mode, rule-inferred timing parameters modulate a PWM signal responsible for switching the inspiration–expiration states of the virtual lung–ventilator system.

The resulting dynamic flow (*Q*) is processed through a numerical integrator module to calculate *V*_c_ across simulation time emulating the physical relationship derived from *V*_c_ =∫ *Q*(*t*) *dt* [5]. As an initial proof-of-concept modeling approach, this integrator operates without a periodic reset condition, resulting in an accumulating volume trace rather than a breath-by-breath tidal volume referenced directly to the functional residual capacity.

The system exports and graphs pressure, flow, and volume curves in real time. These outputs support therapy re-evaluation and clinical interpretation, allowing users to verify physiological coherence and prevent risks prior to clinical implementation.

### 2.2. System Implementation in MATLAB/Simulink

This section details the implementation of the proposed model in MATLAB/Simulink. This simulation environment offers the advantage of specialized toolboxes, such as the Fuzzy Logic Toolbox [23], and the MATLAB and Simulink App Designer [24] for developing graphical applications.

#### 2.2.1. Simulink Model

**Figure 7:**
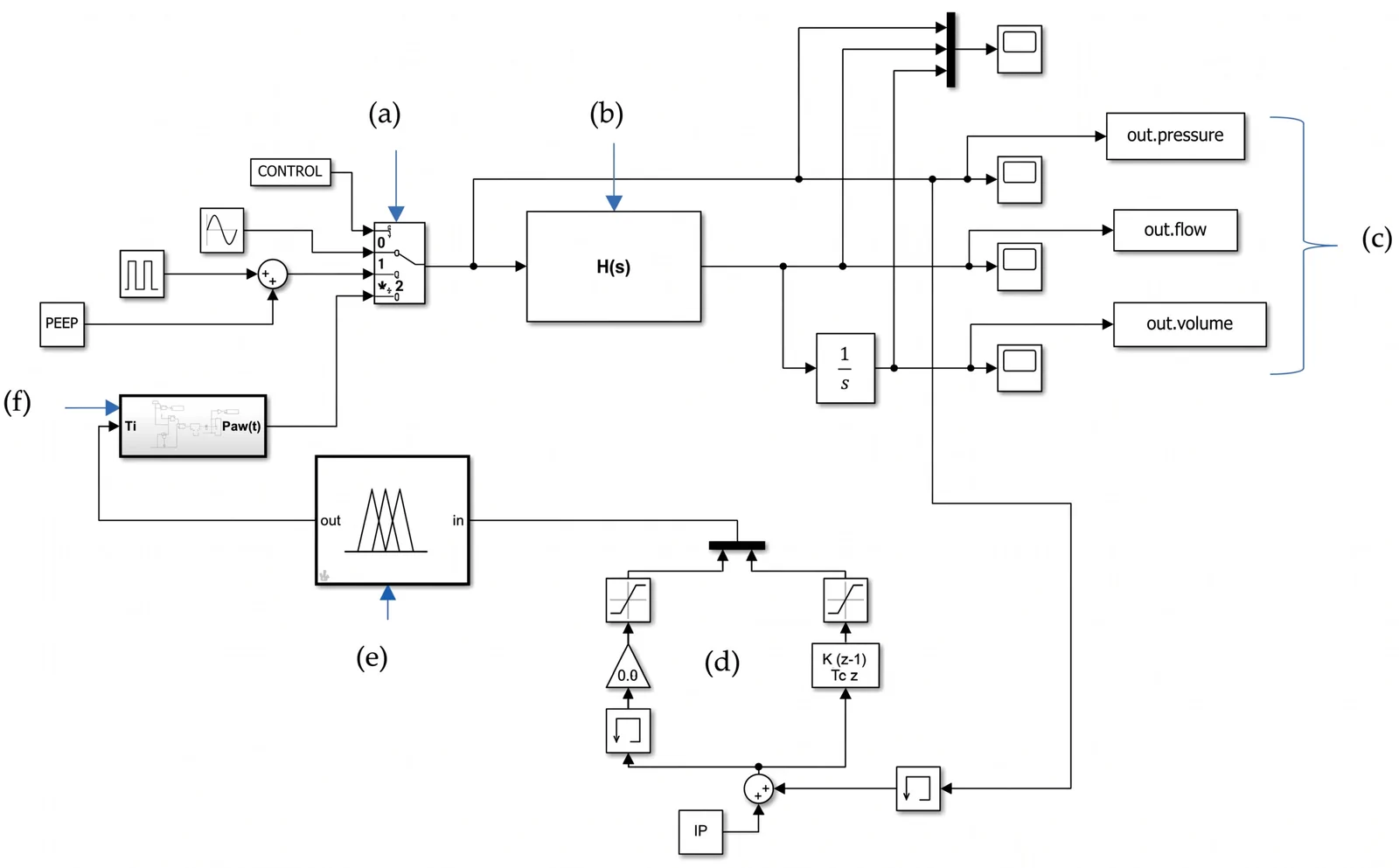
Block diagram in Simulink.

The block diagram of the final solution implemented in Simulink is shown in Figure 7, which features the feedforward architecture of the patient model coupled with the FIS. It consists of six fundamental parts, labeled Figure 7 (a)–(f).

**Figure 8:**
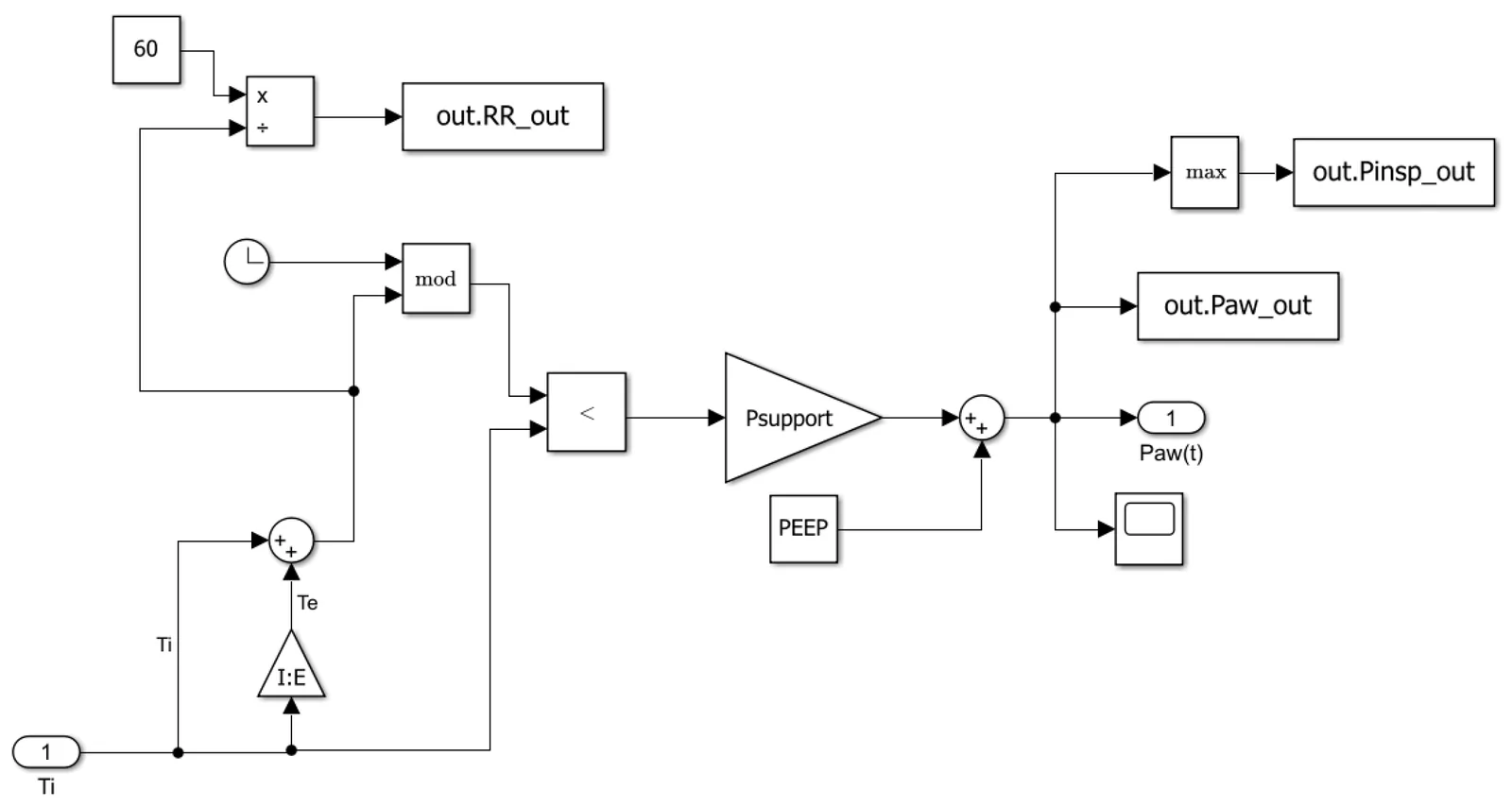
PWM signal generation system.

(a) Input signal selection: Filtering through a Multiport Switch block for different types of signals (sinusoidal, square, and PWM signals) based on the selected mode.
(b) Lung model: Transfer function that models the mechanical behavior of the simulated patient’s lungs.
(c) Output curve visualization: Oscilloscopes are used to visualize respiration signals in real time (pressure–time, volume–time, and airflow–time).
(d) Pressure error calculation: *P*_e_ and Δ*P*_e_ are calculated from the waves generated by the system. Specifically, *P*_meas_ utilized for this calculation is derived directly from the active airway pressure output of the variable pulse-width modulation subsystem, ensuring that the fuzzy controller continuously evaluates deviations between *P*_insp_ and the practically delivered pressure waveform. Within the discrete derivative implementation (Figure 7 (d)), the system utilizes a discrete-time derivative block with an inherited sample time, a proportional gain of 0.8, and clamping saturation limits to prevent derivative kick during sudden pressure transitions.
(e) Fuzzy controller: Implementation of the FIS, which emulates a pressure-controlled mechanical ventilator. It maps *T*_i_ values based on its pressure inputs (*P*_e_ and Δ*P*_e_) across preconfigured profiles.
(f) Variable pulse-width generation subsystem (shown in detail in Figure 8): This block is responsible for modulating the model parameters based on the FIS’s output (*T*_i_). As detailed in Figure 8, temporal coordination is governed by a clock and modulo-based time comparison structure, ensuring that the rule-derived *T*_i_ dictates the switching threshold of the active-duty cycle within each periodic respiratory window, where *TCT* is dynamically computed following Equation (3). Rather than relying on a fixed discrete sampling interval or a hard saturation clock ceiling, the periodic respiratory window coordinates the active *T*_i_ along with the *T*_e_ from the selected *I*:*E* ratio multiplier block, with execution steps synchronized directly with the simulation solver’s temporal integration steps. The resulting control signal, after being scaled by *P*_support_ and added to *PEEP*, determines the *P*_aw_ applied to the patient model. For data processing and results analysis, the pressure, flow, volume, *P*_insp_, *P*_e_, and *RR* variables are exported to the MATLAB workspace in timeseries format.

To ensure dimensional consistency within the feedforward architecture, all pressure variables utilized in the structure of the diagram shown in Figure 8—specifically, *P*_insp_ and *P*_meas_—are strictly comparable, sharing identical units of cmH_2_O. Volume outputs are integrated separately for visualization and analysis and are not mixed into the pressure-error calculation, thereby maintaining dimensional validity. Δ*P*_e_ is a rate of change per discrete step; therefore, its units are cmH_2_O/s. Table 8 summarizes all primary control signals, their corresponding descriptions, and their operational units.

To evaluate the operational performance across all simulated profiles, custom MATLAB post-processing scripts were utilized to extract and quantify steady-state metrics. Following a 40 s simulation run designed to eliminate initial transient responses, time-series data for pressure, airflow, and cumulative volume were isolated by focusing strictly on the final 15 s steady-state window (*t* ≥ max(*t*) − 15 s). Nominal *T*_i_ and *T*_e_ durations were computed based on the total cycle time and configured *I*:*E* ratios. Breath-by-breath cumulative volumes (*V*_c_) and end-expiratory flows (*Q*_e_) were quantified using local peak and trough detection algorithms (findpeaks) to assess volume expansion and dynamic hyperinflation. Finally, pressure errors (*P*_e_) and their discrete derivatives (Δ*P*_e_) relative to the target setpoint were averaged to compute the mean and standard deviation statistics across the established steady-state interval.

#### 2.2.2. Graphical User Interface

**Figure 9:**
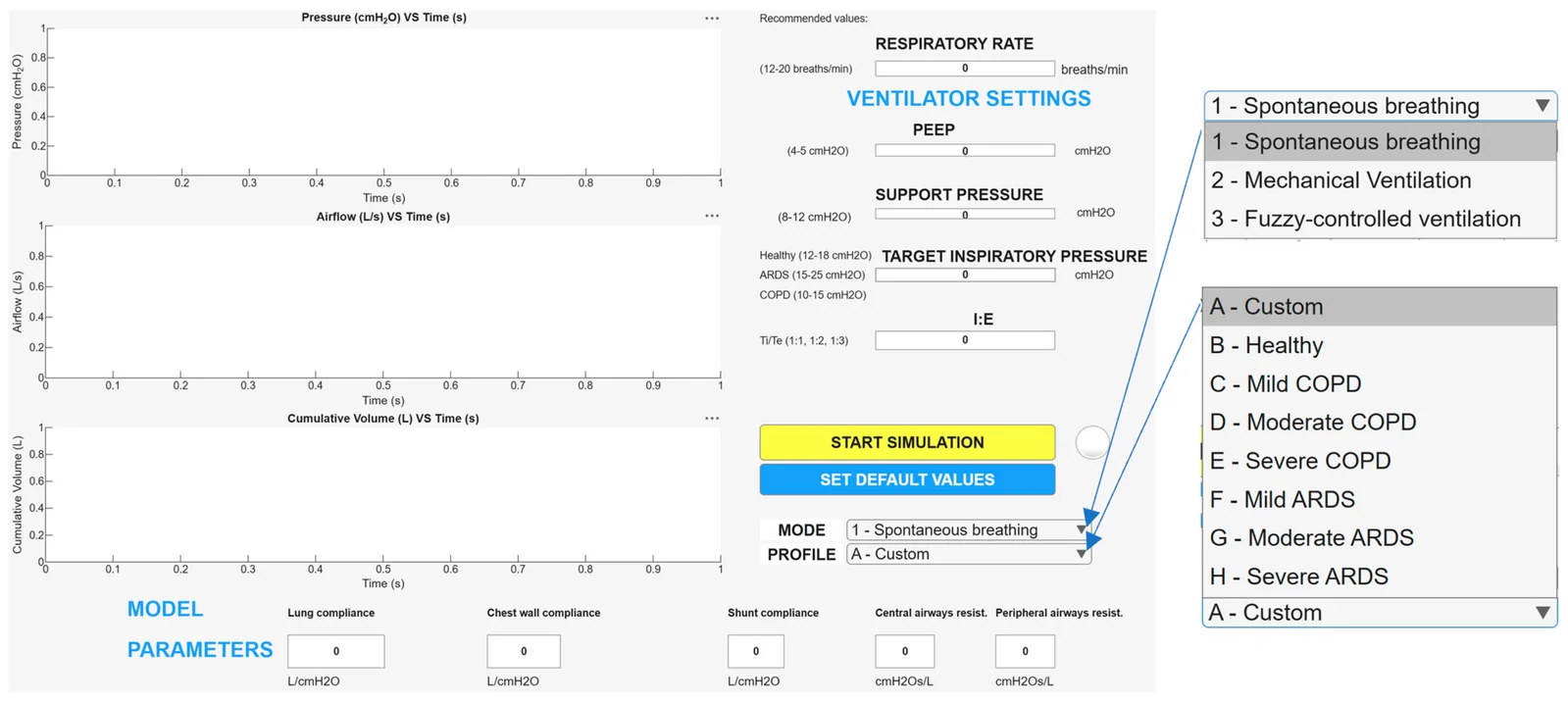
VENTILA2’s GUI with the operating mode and respiratory profile dropdown menus.

The application architecture, the interface of which is illustrated in Figure 9, was developed using MATLAB/Simulink’s App Designer [24]. The GUI design contains configurable fields for the customized setup of the simulated respiratory system’s mechanical properties with an option for initialization using preconfigured values (*Set Default Values*).

The selection of pathophysiological conditions and ventilatory strategies is managed through dynamic dropdown menus, allowing the user to configure system parameters prior to simulation execution. This modular design facilitates smooth switching between different clinical scenarios, optimizing the assessment of patient–ventilator interaction.

The desired respiratory profile is established by assigning discrete values to the mechanical variables of central resistance (*R*_C_), peripheral resistance (*R*_P_), and the compliances of the lungs (*C*_L_), chest wall (*C*_W_), and collateral structures (*C*_S_), emulating healthy physiological states or pathological alterations (COPD and ARDS) stratified by severity levels. Simultaneously, the parameters required by the selected mode must be configured, among which one can find the *RR*, *PEEP*, *P*_support_, *P*_insp_, and the *I*:*E* ratio. In the case of using custom values, there is a specific option for this purpose (*Custom*).

Another key functionality of the GUI is the integrated visualization of the patient’s response using the respiratory curves from the last simulation (temporal evolution of pressure, volume, and flow), so that it can serve the clinician as a decision-making support tool regarding the maintenance or need to redesign the therapy for a specific patient.

## 3. Case Studies

To demonstrate how VENTILA2 works, we present two case studies that illustrate the platform’s ability to replicate physiological and pathological scenarios (severe ARDS and moderate COPD) managed by the FIS (Mode 3). Each case displays the real-time pressure, flow, and volume waveforms, providing an intuitive view of the interaction between the ventilator and the respiratory system.

While the VENTILA2 framework is architecturally designed to support eight pathological gradations, two representative clinical scenarios—severe ARDS and moderate COPD—were selected for detailed evaluation. These profiles represent the physiological extremes of pulmonary mechanics (severe restrictive impairment with rapid time constants versus obstructive airflow limitation with markedly prolonged expiratory time constants). Consequently, assessing the rule-based fuzzy system across these boundary conditions provides an evaluation of its output-mapping consistency without requiring redundant iteration through intermediate severity tiers.

Each physiopathological scenario was simulated for a total duration of 40 s with a fixed time step to ensure complete dissipation of initial transient responses, allowing the system to achieve a stable periodic steady state prior to data extraction.

### 3.1. Stage I: Model Configuration

The VENTILA2 platform employs a bicompartmental model to simulate the heterogeneous mechanical behavior of the respiratory system modeled by two distinct chambers characterized by specific resistance and compliance values [5,15]. This configuration dictates the lung’s response to the pressure delivered by the ventilator, directly influencing the rate of alveolar filling and emptying during the inspiratory (*T*_i_) and expiratory (*T*_e_) phases [4]. In the severe ARDS model, the marked reduction in pulmonary compliance decreases the respiratory system’s expiratory time constant ($\tau_{e}=R_{T}\cdot C_{T}$, where $R_{T}$ = $R_{C}$ + $R_{P}$), driving a higher peak expiratory flow governed by driving pressure and resistance, while the altered compliance dictates the decay rate [5,17]. Conversely, in the moderate COPD scenario, the increased peripheral airway resistance hinders complete lung emptying, requiring precise adjustments to *T*_e_ to manage the respiratory cycle effectively [25].

To evaluate the platform’s performance across these different clinical scenarios, specific parameter value sets are defined in Table 4.

### 3.2. Stage II: Model Construction

The transfer functions derived for each clinical profile, obtained by substituting the respiratory model parameter values stated in Table 4 into the model’s transfer function (7), are presented below: Equation (11) corresponds to the severe ARDS scenario, while (12) represents the moderate COPD case.

(11) $$H_{1}\left(s\right)=\frac{s^{2}+30s}{s^{2}+230s+6000}$$

(12) $$H_{2}\left(s\right)=\frac{s^{2}+0.321s}{s^{2}+200.321s+64.197}$$

### 3.3. Stages III and IV: Fuzzy Inference, Curve Generation and Decision Making

**Figure 10:**
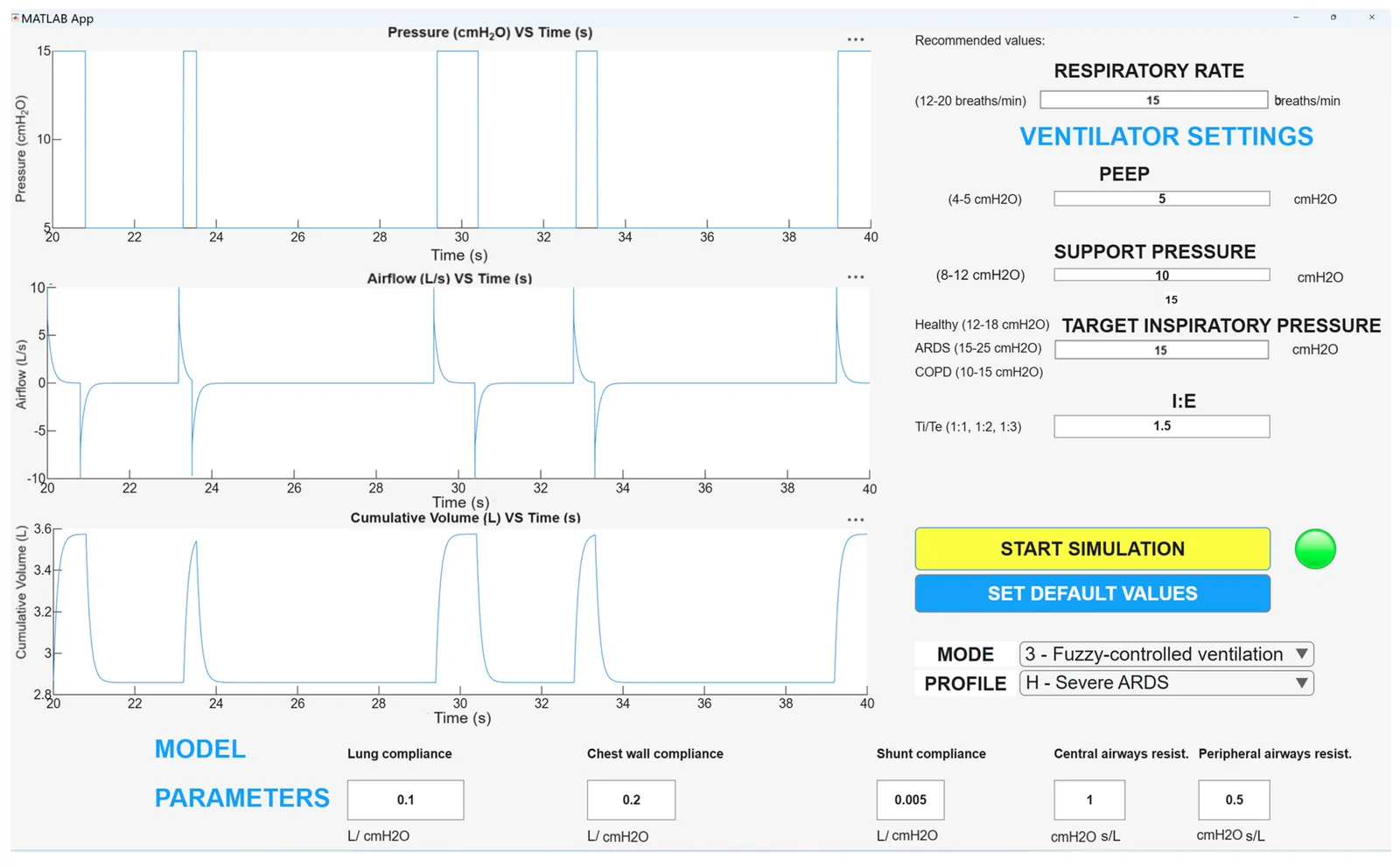
First case study (severe ARDS).

The first case study, shown in Figure 10, corresponds to a mechanically ventilated patient with severe ARDS (*RR* = 15 breaths/minute, *PEEP* = 5 cmH_2_O, *P*_support_ = 10 cmH_2_O, *P*_insp_ = 15 cmH_2_O, and *I*:*E* = 1:1.5). In this scenario, the pressure signal exhibits a square waveform with a variable pulse width, determined by the rule-mapped *T*_i_ derived from the FIS. Note that *P*_insp_ (15 cmH_2_O) serves strictly as the target setpoint reference for the FIS to compute the pressure error (*P*_e_), whereas the sum of *PEEP* and *P*_support_ (15 cmH_2_O) dictates the actual physical amplitude of the square pressure waveform applied to the model.

The observed mechanical response is intrinsically conditioned by the pathophysiology of ARDS: the marked reduction in pulmonary compliance leads to a significantly shorter passive expiratory time constant ($\tau_{e}=\left(R_{C}+R_{P}\right)\cdot C_{T}\approx 0.100\;s$). Because the series two-compartment model is a second-order system, this single lumped $\tau_{e}$ serves as a clinical approximation rather than the system’s exact analytical response. However, it effectively demonstrates the accelerated lung emptying (a faster expiratory flow) compared with a healthy physiological state, without modifying the total expiratory phase duration ($T_{e}$) allocated by the ventilator cycle [17,25]. The FIS maps this complex interaction, translating the preconfigured mechanical requirements of the injured lung into corresponding $T_{i}$ values, while the volume reflects an oscillating behavior with cycle-to-cycle variability, faithfully simulating the characteristic dynamics of patient–ventilator interaction in severe pathology states [4,11].

**Figure 11:**
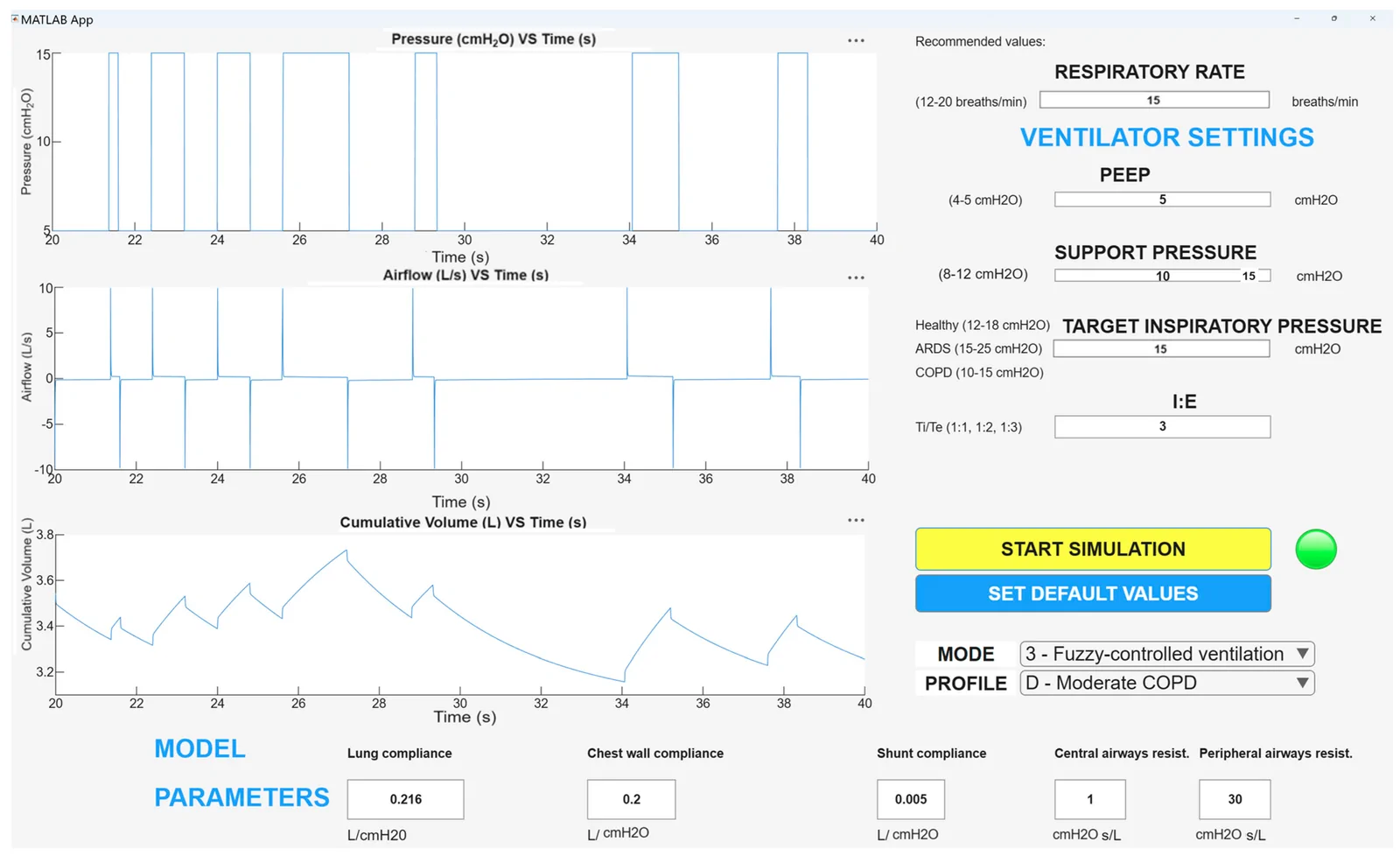
Second case study (moderate COPD).

In the second case study, shown in Figure 11, a patient with moderate COPD is considered, coupled to a mechanical ventilator with fuzzy control (*RR* = 15 breaths/minute, *PEEP* = 5 cmH_2_O, *P*_support_ = 10 cmH_2_O, *P*_insp_ = 15 cmH_2_O, and *I*:*E* = 1:3). This scenario represents an intermediate peripheral obstruction, characterized by significantly elevated flow resistance (*R*_P_ = 30 cmH_2_O·s/L) [26]. Unlike the previous case, expiratory flow limitation is the dominant factor; in the simulation, it is observed that the flow does not return completely to baseline before the start of the next cycle due to the prolonged time constant ($\tau_{e}=(R_{C}$ + $R_{P})\cdot C_{T}\approx 3.22\;s$), faithfully reproducing the phenomenon of air trapping or auto-PEEP [25,26]. This dynamic hyperinflation manifests in the volume curve as a progressive upward shift, evidencing the lung’s inability to empty completely between breaths [17]. In parallel, the pressure signal displays irregular square variations, reflecting the rule-based system’s response within this obstructive ventilatory mechanics regime.

### 3.4. Quantitative Operational Verification

To demonstrate the internal operational performance and consistency of the proposed framework, quantitative evaluations were performed across all seven predefined physiopathological scenarios under uniform baseline ventilatory conditions (utilizing a fixed *P*_insp_ = 15 cmH_2_O and *I*:*E* = 1:2, with a theoretical *RR* set to 15 breaths/min, resulting in nominal *T*_i_ = 1.00 s and *T*_e_ = 2.00 s). The simulation time was set to 40 s.

Table 9 summarizes the resulting operational metrics extracted from the simulation runs. The evaluated parameters include the mean and standard deviation of steady-state *P*_e_, resulting estimated *V*_c_, and end-expiratory flow (*Q*_e_) to quantify dynamic hyperinflation and auto-PEEP in COPD profiles.

As detailed in Table 9, cumulative volumes (*V*_c_) show pronounced degradation patterns. In ARDS profiles, *V*_c_ decreases progressively from mild (0.67 ± 0.20 L) to severe (0.60 ± 0.15 L) due to the intrinsic loss of lung compliance (stiffening pulmonary tissue), which restricts volume expansion under a constant pressure profile. Similarly, obstructive severity in COPD results in a sharp reduction in delivered volume—dropping from 0.50 ± 0.44 L in mild COPD down to 0.20 ± 0.20 L in severe COPD—alongside elevated pressure tracking errors reaching up to 8.71 ± 3.35 cmH_2_O, reflecting the feedforward architecture.

Effective timing and frequency metrics remain completely invariant across all simulated pathologies. The system maintains a generated inspiratory time of 1.33 ± 0.02 s, a passive expiratory time of 2.67 ± 0.03 s, and an effective respiratory rate of *RR* = 13.1 ± 1.97 breaths/min. This demonstrates that the controller architecture maintains fixed temporal boundaries rather than dynamically adapting phase durations to individual disease states.

## 4. Discussion

The proposed work represents an advancement by integrating multicompartmental models, fuzzy control, and the specific simulation of pathologies categorized by severity. The two-compartment model effectively represents pulmonary heterogeneity. While the platform serves as a useful in silico tool for evaluating ventilatory configurations, it remains a preclinical proof of concept.

Rather than functioning as a real-time closed-loop feedback tracking controller [10,27], the system operates as a scenario-based, feedforward parameter-mapping framework that translates specific respiratory mechanics into optimized temporal and pressure configurations. Nevertheless, its modular architecture allows for future expansion, whether through the incorporation of new pathologies via preconfigured values or the adaptation of the transfer function to other respiratory mechanics models. Furthermore, pursuing external validation is currently a priority.

The core of the proposed system centers on the implementation of the fuzzy logic-based controller. While classical control approaches such as Proportional-Integral-Derivative (PID) control, implemented by Tamburrano et al. [10], and advanced control frameworks like Model Predictive Control (MPC), implemented by D’Orsi et al. [6], are well established, the main advantage of implementing a Mamdani-type FIS, implemented by Güler et al. [4] and VENTILA2, lies in its theoretical capacity to handle nonlinear biological responses and integrate heuristic clinical rules through linguistic rules and MFs without requiring precise mathematical modeling or high computational loads [19,28].

Our proposal manages the uncertainty inherent in routine clinical measurements while acting as a conceptual framework to assist user interpretation. By structuring the logic into an explicit rule base, the system functions as an interpretable interface that emulates clinical reasoning. This computational flexibility enables the platform to handle imprecise parameters and map adjustments to variations in respiratory mechanics, providing a profile-driven, adaptable simulation that responds flexibly to changes in patient status [4].

Compared with the previous literature (Table 10), VENTILA2 advances beyond the isolated use of compartmental modeling or fuzzy control. While prior studies typically focus on healthy archetypes or a single static condition, this work integrates a continuous spectrum of multi-severity respiratory profiles—spanning mild to severe COPD and ARDS—within an interactive graphical environment coupled with a fuzzy logic controller. Consequently, the platform provides a comprehensive, scalable framework that captures inter-patient variability across a wide clinical spectrum rather than isolated data points.

Regarding the physiological representation of the respiratory system, two mathematical modeling approaches for the respiratory system can be distinguished. On the one hand, there is the compartmental approach, used by Jaber et al. [11] and Güler et al. [4], which segments the respiratory tract into chambers with homogeneous properties. On the other hand, there is the pneumatic approach, such as that of Tamburrano et al. [10], where the Simscape Fluids library is used to emulate the electromechanical interaction of the internal components of the mechanical ventilator (valves and other hardware).

One of the most innovative aspects of the proposed system is the recreation of pathophysiological scenarios stratified by their degree of severity. Jaber et al. [11] and Tamburrano et al. [10] evaluate ventilator performance without considering specific profiles. D’Orsi et al. [6] advanced this by including healthy, COPD, and ARDS profiles using flow resistance and compliance values adjusted by 50% from their nominal physiological baselines. Our proposal expands this scope by offering a range of clinical severity that allows for the evaluation of the intelligent controller’s response to disease progression.

Validation remains a significant challenge for clinical simulators. Güler et al. [4] validated their fuzzy system in vivo using an animal model (Wistar rats), demonstrating that their controller responded in a practical and realistic manner to changes in resistance. Tamburrano et al. [10] opted for a robust quantitative validation, comparing their simulation performance directly with real waveform data extracted from the literature to verify the behavior of volume-controlled and pressure-controlled ventilation modes. In contrast, Jaber et al. and D’Orsi et al. [6] focused on purely numerical or virtual validations.

Overall, our proposed work fills an evident technological and clinical gap in the literature. While previous studies offer highly precise mechanical models with classical control—such as Tamburrano et al. [10]—or apply fuzzy control only in basic 1- or 2-compartment anatomical models—such as Güler et al. [4]—the present proposal combines fuzzy control with a multi-compartment model capable of simulating COPD and ARDS at different levels of clinical severity. This establishes a simulation framework much closer to the true demands and variability of NIV in ICUs and IRCUs.

### Limitations and Future Research Lines

The scope of the proposed system is constrained by its strictly in silico nature and the lack of clinical validation through direct comparison with real-world, bedside data. Specifically, the platform relies on predefined synthetic respiratory parameters, simplified waveforms, and a linear two-compartment model without direct patient-level or bedside ventilator data validation. Furthermore, it uses a profile-configured feedforward architecture rather than a true physiological closed-loop feedback loop where patient-model responses dynamically update controller parameters in real time [29].

The adoption of a linear compartmental lung model based on resistive–capacitive (RC) electrical analogies is efficient for low respiratory frequencies, yet it omits the airflow inertial term, which gains clinical relevance in high-frequency ventilation conditions [7]. Likewise, the proposal idealizes the patient–ventilator interaction and is limited to the pressure-controlled ventilation (PCV) mode, which is one of the two most common modes alongside volume-controlled ventilation (VCV) [10].

Parametrization presents a challenge regarding the difficulty of identifying the exact mechanical properties of a specific patient. Additionally, while disease severity is stratified through peripheral resistance (*R*_P_) and *C*_L_, central airway resistance (*R*_C_), *C*_W_, and *C*_S_ were maintained at constant baseline values across the evaluated case studies (specifically, severe ARDS and moderate COPD). In real clinical scenarios, these secondary parameters also exhibit inter-patient variability. Although their physiological fluctuation is narrower compared with parenchymal compliance or peripheral obstruction, this simplification means absolute pressure tracking errors and settling behaviors may be subtly influenced.

Given the profound variability in users of NIV devices [1], values such as resistance and compliance require dynamic identification from routine clinical data [1]. Furthermore, certain parameter settings evaluated in this study—such as a driving pressure of 10 cmH_2_O applied to severe ARDS compliance profiles—result in cumulative volumes that exceed strict lung-protective ventilation targets (≤6 mL/kg) [17], highlighting that the current architecture lacks automated volumetric safety constraints. Future iterations of VENTILA2 must integrate hard saturation constraints, specifically, automated volume- and pressure-limiting safety loops, to preclude simulated volutrauma and fully align with modern lung-protective guidelines for ARDS.

For future research, it will be crucial to carry out retrospective and prospective validation with external patient cohorts (or trials with virtual cohorts) to adapt the tool to medical routine and evaluate its real-world performance. To this end, we propose the integration of real data from clinical ventilators using clinical waveforms (pressure, flow, volume) collected in intermediate respiratory care units or intensive care units to calibrate the mathematical model.

Additionally, we propose increasing the complexity of the model by incorporating ventilatory modalities that were beyond the scope of this initial work, such as VCV or hybrid modes, as well as the development of nonlinear lung models and structures that account for inertia for rapid ventilation scenarios [5]. The development of machine learning algorithms may also allow for the classification of patients as stable vs. unstable, strengthening clinical decision making based on a real-time feature analysis of the synthetic patient’s response.

Furthermore, regarding the design of the fuzzy controller, formal quantitative testing for sensitivity to changes in membership function boundaries was not performed in this preliminary phase nor were other MF shapes (such as trapezoidal or gaussian) tested, representing an important avenue for future methodological refinement. Also, the assumption of an ideal square wave with sudden pressure transitions instead of a trapezoidal signal does not faithfully capture the real ventilator dynamics. This simplification introduces a margin of error regarding the crispness of the pressure error gradient during the early inspiratory phase, which may slightly overestimate the controller’s mapping speed.

To rigorously benchmark the performance advantages of the fuzzy controller, future work will implement a standardized comparative experimental setup. This framework will ingest real-world, time-series ventilator waveforms (pressure, flow, and volume) captured from patients undergoing mechanical ventilation in ICU environments via retrospective clinical databases. Using these synchronized patients’ datasets as common reference inputs across simulation runs, VENTILA2’s scenario-based mapping will be directly evaluated against traditional PID controllers and advanced MPC architectures under identical pathological boundary conditions.

Lastly, we plan to expand our platform into a fully developed intelligent clinical CDSS upon hardware-in-the-loop bench testing and external retrospective validation using clinical databases. The system will aim to assist healthcare personnel in adjusting NIV settings within acute care environments such as IRCUs or ICUs.

## 5. Conclusions

This work successfully demonstrates the feasibility of an in-silico platform for the pathology-specific management of NIV, successfully transitioning from trial-and-error clinical practices to a structured, model-based approach. By integrating a Mamdani-type fuzzy controller within a bicompartmental pulmonary model, the proposed system provides a framework for simulating preconfigured mechanical interactions in both healthy and pathological states (COPD and ARDS stratified from mild to severe).

The primary contribution of this platform lies in its interpretability and the safety it affords, allowing for the predictive evaluation of ventilatory strategies without the inherent risks of bedside experimentation. While this proof of concept establishes baseline internal quantitative verification across all simulated profiles, future research will focus on expanding the pathological dataset and realizing direct benchmark comparisons against other control strategies like PID and MPC. Furthermore, future developments must incorporate strict hardware and software safety constraints—such as hard saturation volume- and pressure-limiting loops—to prevent simulated volutrauma. Such extensions will be critical in evolving this prototype from a research-oriented simulation framework into a reliable, bedside clinical decision support system, following rigorous retrospective and prospective external validation using real clinical ventilator waveforms.

## Figures and Tables

**Table 2:** Ventilation control parameters.

Parameter	Utility	Units
Respiratory rate (*RR*)	Crucial to construct the pressure signal	Breaths/minute
Positive end-expiratory pressure (*PEEP*)	Required for modeling the pressure signal amplitude	cmH_2_O
Target inspiratory pressure (*P*_insp_)	Used as a reference for fuzzy estimation	cmH_2_O

**Table 3:** Physiological model parameters.

Parameter	Units
Lung compliance (*C*_L_)	L/cmH_2_O
Chest wall compliance (*C*_W_)	L/cmH_2_O
Shunt compliance (*C*_S_)	L/cmH_2_O
Total compliance (*C*_T_)	L/cmH_2_O
Central airways resistance (*R*_C_)	cmH_2_O/(L/s)
Peripheral airways resistance (*R*_P_)	cmH_2_O/(L/s)

**Table 4:** Mechanical parameters of the respiratory model for the different respiratory profiles.

Clinical Profile	*R*_C_ (cmH_2_O/(L/s))	*R*_P_ (cmH_2_O/(L/s))	*C*_L_ (L/cmH_2_O)	*C*_W_ (L/cmH_2_O)	*C*_S_ (L/cmH_2_O)
Healthy	1.00	0.50	0.20	0.20	0.005
Mild COPD	1.00	5.00	0.21	0.20	0.005
Moderate COPD	1.00	30	0.216	0.20	0.005
Severe COPD	1.00	50	0.218	0.20	0.005
Mild ARDS	1.00	0.50	0.13	0.20	0.005
Moderate ARDS	1.00	0.50	0.11	0.20	0.005
Severe ARDS	1.00	0.50	0.10	0.20	0.005

**Table 5:** Membership function breakpoints for the two antecedents (*P*_e_ and Δ*P*_e_). Units are cmH_2_O and cmH_2_O/s, respectively.

Fuzzy Set	First Antecedent (*P*_e_)	Second Antecedent (Δ*P*_e_)
NB	[−40, −40, −20]	[−10, −10, −5]
NS	[−40, −20, 0]	[−10, −5, 0]
Z	[−20, 0, +20]	[−5, 0, +5]
PS	[0, +20, +40]	[0, +5, +10]
PB	[+20, +40, +40]	[5, +10, +10]

**Table 6:** Membership function breakpoints for the consequent (*T*_i_). Units are seconds (s).

Fuzzy Set	Consequent (*T*_i_)
INS1	[0, 0, 1]
INS2	[0, 1, 2]
INS3	[1, 2, 3]
INS4	[2, 3, 4]
INS5	[3, 4, 5]
INS6	[4, 5, 6]
INS7	[5, 6, 6]

**Table 7:** Fuzzy conjunctive rules.

$P_{e}(k)/\Delta P_{e}(k)$	NB	NS	Z	PS	PB
NB	INS1	INS1	INS2	INS3	INS4
NS	INS2	INS2	INS3	INS4	INS5
Z	INS2	INS3	INS4	INS5	INS6
PS	INS3	INS4	INS5	INS6	INS7
PB	INS4	INS5	INS6	INS7	INS7

**Table 8:** Summary of control signals, descriptions, and dimensional units.

Signal/Variable	Description	Units
Airway opening pressure (*P*_aw_)	Total pressure applied to the upper airway by the ventilator.	cmH_2_O
Target inspiratory pressure (*P*_insp_)	Reference setpoint pressure used by the fuzzy controller.	cmH_2_O
Measured airway pressure (*P*_meas_)	Active pressure sampled from the PWM modulation subsystem.	cmH_2_O
Pressure error (*P*_e_)	Difference between target and measured pressure (*P*_insp_ − *P*_meas_).	cmH_2_O
Change in pressure error (Δ*P*_e_)	Rate of change in the pressure error between consecutive steps.	cmH_2_O/s
Airflow (Q)	Dynamic air exchange rate though the respiratory system.	L/s
Cumulative volume (*V*_c_)	Integrated volumetric accumulation resulting from airflow.	L
Inspiratory time (*T*_i_)	Rule-mapped duration of the inspiratory phase.	s

**Table 9:** Operational metrics across the respiratory profiles.

Clinical Profile	*P*_e_ (cmH_2_O)	*V*_c_ (L)	*Q*_e_ (L/s)
Healthy	8.71 ± 3.35	0.79 ± 0.29	−8.72 ± 1.73
Mild COPD	8.65 ± 3.41	0.50 ± 0.44	−9.39 ± 0.53
Moderate COPD	8.64 ± 3.43	0.23 ± 0.08	−9.78 ± 0.02
Severe COPD	6.43 ± 4.80	0.20 ± 0.20	−9.85 ± 0.01
Mild ARDS	8.71 ± 3.35	0.67 ± 0.20	−8.97 ± 1.50
Moderate ARDS	8.71 ± 3.35	0.62 ± 0.17	−9.07 ± 1.38
Severe ARDS	8.71 ± 3.35	0.60 ± 0.15	−9.13 ± 1.31

**Table 10:** Comparative study of previous research works and our proposal according to five specific criteria.

Study	Software	Mathematical Model	Pathologies	Control Strategy	Validation
Jaber et al. (2020) [11]	MATLAB/Simulink	Bicompartmental	n/a	n/a	Numerical simulation
Güler et al. (2014) [4]	MATLAB/Simulink and LabView	Single compartment	n/a	Mamdani fuzzy control	In vivo (Wistar rats)
Tamburrano et. al. (2022) [10]	MATLAB/Simulink (Simscape Fluids)	Pneumatic (hardware level)	n/a	Traditional PID	Quantitative waveform comparison
D’Orsi et al. (2024) [6]	MATLAB/Simulink	Compartmental	COPD and ARDS (static 50% adjustment)	MPC	Numerical simulation
Our proposal	MATLAB/Simulink	Bicompartmental series	COPD and ARDS (multi-severity levels)	Mamdani fuzzy control	Preclinical proof-of-concept simulation

## Data Availability

This study was based on computational simulation and did not involve patient data or external datasets. The computational materials developed for this proof of concept, including the Simulink model, the fuzzy rule base, and the prototype application, are not currently publicly available because VENTILA2 remains under active development.

## Abbreviations

The following abbreviations are used in this manuscript:

*C*_L_	Pulmonary compliance
COPD	Chronic obstructive pulmonary disease
*C*_S_	Shunt compliance
*C*_T_	Total compliance
*C*_W_	Chest wall compliance
CDSS	Clinical decision support system
FIS	Fuzzy inference system
GUI	Graphical user interface
ICU	Intensive care unit
*I*:*E*	Inspiratory-to-expiratory ratio
IRCU	Intermediate respiratory care unit
MF	Membership function
MPC	Model predictive control
NIV	Non-invasive ventilation
*P*_aw_	Airway opening pressure
PCV	Pressure-controlled ventilation
Δ*P*_e_	Change in pressure error
*P*_e_	Pressure error
*PEEP*	Positive end-expiratory pressure
PID	Proportional-Integral-Derivative
*P*_insp_	Target inspiratory pressure
*P*_meas_	Measured pressure
*P*_support_	Support pressure
PWM	Pulse-width modulation
*Q*	Airflow
*Q*_e_	End-expiratory flow
*R*_C_	Central airways resistance
*R*_P_	Peripheral airways resistance
*RR*	Respiratory rate
ARDS	Acute respiratory distress syndrome
*TCT*	Total cycle time
*T*_e_	Expiratory time
*T*_i_	Inspiratory time
*T*_S_	Discrete sample time
*τ*_e_	Expiratory time constant
*V*_c_	Cumulative volume
VCV	Volume-controlled ventilation
